# Epigenetic Aging and Colorectal Cancer: State of the Art and Perspectives for Future Research

**DOI:** 10.3390/ijms22010200

**Published:** 2020-12-28

**Authors:** Andrea Maugeri, Martina Barchitta, Roberta Magnano San Lio, Giovanni Li Destri, Antonella Agodi, Guido Basile

**Affiliations:** 1 Department of Medical and Surgical Sciences and Advanced Technologies “GF Ingrassia”, University of Catania, via S. Sofia, 87, 95123 Catania, Italy; andrea.maugeri@unict.it (A.M.); robimagnano@gmail.com (R.M.S.L.); glides@unict.it (G.L.D.); agodia@unict.it (A.A.); 2 Department of General Surgery and Medical-Surgical Specialties, University of Catania, via S. Sofia, 78, 95123 Catania, Italy; gbasile@unict.it

**Keywords:** DNA methylation, microRNA, biological aging, chronic diseases

## Abstract

Although translational research has identified a large number of potential biomarkers involved in colorectal cancer (CRC) carcinogenesis, a better understanding of the molecular pathways associated with biological aging in colorectal cells and tissues is needed. Here, we aim to summarize the state of the art about the role of age acceleration, defined as the difference between epigenetic age and chronological age, in the development and progression of CRC. Some studies have shown that accelerated biological aging is positively associated with the risk of cancer and death in general. In line with these findings, other studies have shown how the assessment of epigenetic age in people at risk for CRC could be helpful for monitoring the molecular response to preventive interventions. Moreover, it would be interesting to investigate whether aberrant epigenetic aging could help identify CRC patients with a high risk of recurrence and a worst prognosis, as well as those who respond poorly to treatment. Yet, the application of this novel concept is still in its infancy, and further research should be encouraged in anticipation of future applications in clinical practice.

## 1. Introduction

Colorectal cancer (CRC) is considered to be the second-most and the third-most common cancer among men and women, respectively, accounting for 9.7% of all cancers worldwide. Along with breast and lung cancer, CRC represents one of the leading causes of cancer death [1]. The age-standardized incidence rate of colorectal cancer is 20.6 per 100,000 individuals in men, and 14.3 per 100,000 individuals in women. Notably, the World Health Organization (WHO) estimates an increase of nearly 80% in new cases and deaths from CRC by 2030 [2]. The majority of CRCs are sporadic (70–80%), and most of these cases are in people over 50 years of age, while only a small proportion are due to inherited forms, either familial adenomatous polyposis or *mutY DNA glycosylase* (*MUTYH*)-gene associated polyposis (<1%); non-polyposis hereditary CRC; or Lynch syndrome (2–5%). Moreover, a specific subgroup of cases (nearly 20%) is represented by those with an associated hereditary component, which has not yet been well established and is known as familial CRC [3]. From a genetic point of view, the first route of CRC carcinogenesis involves the accumulation of mutations that lead to oncogene activation and suppressor gene inactivation [4]. The second route, however, involves the accumulation of errors during DNA replication due to mutations in the genes responsible for its repair [5]. 

However, several factors play an important role in the etiology of CRC, including male sex, inflammatory bowel disease, diabetes, ethnicity, and lifestyle [6]. Specifically, lifestyle factors, such as smoking [7,8], certain dietary habits [6], alcohol intake [9], and increased body weight [10], are considered to be the main risk factors for developing CRC. The etiological and risk factors of CRC development reflect the multifactorial nature of the disease. Interestingly, both environmental exposure and behavior might affect the biological aging process, which is described as an increased state of vulnerability, with cell senescence, mitochondrial dysfunction, genomic instability, epigenetic changes, and telomere erosion as underlying mechanisms [11]. In particular, CRC is characterized by the gradual accumulation of genetic and epigenetic changes, leading to the transformation of normal colonic mucosa into invasive cancer [12]. In fact, a loss of genomic and/or epigenomic stability affects the majority of early neoplastic lesions in the colon, accelerating the activation of oncogenes and the inactivation of tumor suppressor genes [13,14]. In the last decade, translational research has identified a large number of potential biomarkers involved in CRC carcinogenesis. For instance, the neutrophil-to-lymphocyte ratio represents a simple hematological parameter associated with the response to treatment and the prognosis of CRC patients [15,16]. Despite the progresses made, however, a better understanding of the molecular pathways associated with biological aging in colorectal cells and tissues is needed. For instance, previous studies have proposed several approaches to estimate biological aging through assessing the epigenetic changes that occur as a result. The majority of these approaches ae based on DNA methylation and microRNA (miRNA) signatures. 

Here, we aim to point out the state of the art about the role of age acceleration, defined as the difference between epigenetic age and chronological age, in the initiation and progression of CRC. First, we define the role of aging as a risk and prognostic factor of CRC, with a particular focus on the biological events that might characterize its development and progression. Next, we illustrate how DNA methylation drift occurs during colorectal carcinogenesis, summarizing epidemiological studies that use epigenetic age estimators based on different sets of DNA methylation signatures. In the same way, we then describe the changes in miRNA expression that occur with aging, and their association with CRC risk and prognosis. Finally, we discuss the limitations and future perspectives of these findings for the assessment of CRC risks and the management of patients.

## 2. Aging and Colorectal Cancer

In developed countries, the average age at which patients are diagnosed with CRC is 70 years [17]. Indeed, CRC incidence is generally low among people younger than 50 years of age, with a marked and progressive increase with aging. Specifically, most patients with sporadic CRC are older than 50 years of age, while nearly three in four of them are even older than 60 years of age [17]. With respect to prognosis, the survival of CRC patients has slowly but gradually increased in the past decades, reaching nearly 65% in developed countries. It is also observed that relative survival decreases with age, with slight differences by gender—at a younger age, women exhibit a greater survival than men [17]. Early diagnosis of CRC represents a crucial factor to prevent deaths and to improve CRC prognosis. In line, several national and international screening guidelines recommend promoting CRC screening at 50 years of age for individuals at an average risk [1]. In general, screening strategies should adopt annual or bi-annual schemes based on guaiac fecal occult blood or fecal immunochemical tests, sigmoidoscopy every 5 years, or colonoscopy every 10 years [18]. Despite the improvements in these screening options, however, more efforts are needed to develop alternative non-invasive markers, such as blood-based DNA methylation markers or stool DNA tests [19].

In the last decades, it has been observed that age-standardized rates of CRC incidence and mortality vary extremely across different regions of the world [17]. For instance, incidence rates are approximately three-fold higher in developed versus developing countries, while there is less variation in mortality rates. Specifically, the highest incidence rates are reported in Europe, Oceania, Northern America, and Eastern Asia, while they are generally lower in Africa and Southern Asia [17]. For this reason, CRC could be considered as an indicator of socioeconomic development. Indeed, its incidence reflects the epidemiological transition that characterizes some parts of the world [1]. The rising incidence observed in countries undergoing development transition (e.g., Baltic countries, Russia, China, and Brazil) might be linked to a more Westernized lifestyle, which involves unhealthy dietary habits, sedentary life, and obesity [18]. 

The spread of global Westernization might also partially explain the alarming increase in early-onset CRCs over the last decades, which are those tumors diagnosed before the age of 50 years [20]. Because it seems that early-onset tumors are epidemiologically, pathologically, and biologically different than those occurring at an older age, we suppose that other unmodifiable and modifiable risk factors might contribute to the aging process and to the biological dysfunctions that occur with aging. Indeed, biological aging is defined as an increased state of cellular vulnerability, characterized by senescence, mitochondrial dysfunction, genomic and epigenomic instability, and telomere shortening [11]. For this reason, there is a growing need for understanding the biological and molecular events related to the aging process, which often do not reflect the chronological age of an individual. Accordingly, several biomarkers of aging have been proposed to predict biological age, such as metabolites, proteins, inflammation, and clinical factors [21]. In the context of CRC, for example, a combination of physical, biochemical, and hormonal markers was used to estimate biological age among more than 2500 middle-aged South Koreans, 622 of which were diagnosed with CRC [22]. This study suggested that people who exhibited an increased biological age were more likely to develop distal CRC [22]. Beyond this, there are several estimators of biological age, although the most common rely on DNA methylation data. In fact, a recent review of different estimators, based on DNA methylation, telomere length, gene expression, proteins levels, or metabolites, established that DNA methylation-based age is the most promising estimator of biological age [23]. Interestingly, some of these epigenetic age estimators, further discussed below, are generally associated with a risk of death and different diseases [23].

## 3. DNA Methylation Changes as a Function of Age

Several lines of evidence have demonstrated how substantial DNA methylation changes occur with aging [24,25,26]. In normal tissues, the methylation of cytosine-phosphate-guanine (CpG) sites within gene promoters increases with aging, while global DNA methylation levels generally tend to decrease [11,27,28,29,30]. Notably, some of these changes might be associated with a risk for various diseases, including cancer [31,32,33,34,35,36,37]. However, the DNA methylation profile of cancer tissues is profoundly different compared with normal tissues [38], and the changes that occurred depend on the cancer type. De novo methylation of CpG sites, the so-called hypermethylation, typically occurs in tumor suppressor genes and suppresses their functions [38]. A recent comparison between aged and cancer tissues demonstrated that they share some hypermethylated regions with a similar chromatin structure [38]. By contrast, aging and cancer development act differentially on the hypomethylation of the DNA regions [38]. With respect to CRC, a specific CpG island methylator phenotype (CIMP), characterized by the hypermethylation of the genes involved in controlling cell growth and survival, is associated with various molecular and clinicopathological features, as summarized by previous reviews [39,40]. For example, CIMP+ status is more often observed in older patients, females, and high-grade proximal tumors. Moreover, CIMP+ tumors often exhibit mutations in the *BRAF* gene, promoter hypermethylation of the *mutL homolog 1* (*MLH1*) gene, and a deficiency of mismatch repair [41]. Some in vivo studies have investigated whether age-dependent methylation changes in apparently healthy colonic mucosa might be associated with CRC development. For instance, Maegawa and colleagues compared the genome-wide methylation profile of the colon between 3- and 35-month old mice, showing a high rate of both hyper- and hypo-methylation events as a function of age [42]. Interestingly, some of these events also occurred in the lung, liver, and spleen of mice, with a partial conservation between age-related hypermethylation in human and mouse colons [42]. More recently, Tao and colleagues used mouse colon-derived organoids to investigate the role of age-related spontaneous promoter DNA hypermethylation in cancer transformation [43]. Notably, they found that promoter hypermethylation of the genes involved in the Wnt pathway spontaneously arose in cells mimicking the human aging phenotype. The silencing of these genes, in turn, resulted in a stem-like state and differentiation defects [43]. In humans, it is obviously much more difficult to assess whether age-related DNA methylation changes in normal colonic mucosa might be associated with CRC development. Magnani and colleagues, however, compared the methylation levels of 38 tumor suppressor genes and *long interspersed nuclear elements 1* (*LINE-1*) sequences between young (i.e., ≤40 years old) and old (> 60 years old) CRC patients [44]. In general, they found a higher proportion of hereditary CRC forms in the young group, which contrasted with a lower proportion of hypermethylated genes. Thus, their findings suggest that the genetic and epigenetic make-up of carcinomas differ between young and old CRC patients [44].

## 4. DNA Methylation-Based Biological Aging

**Figure 1:**
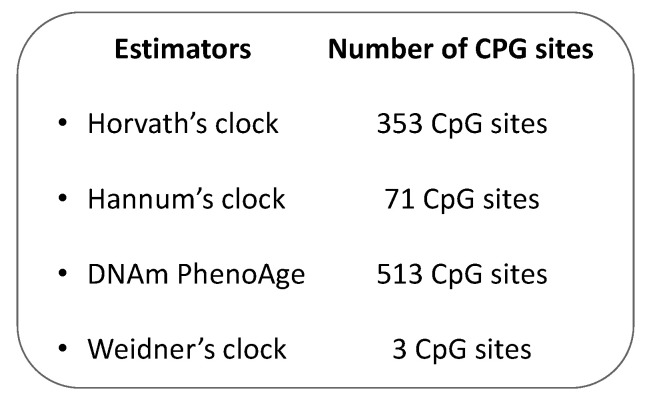
The most common DNA methylation-based biological aging estimators.

In the last decades, several studies have proposed and used different biomarkers, which are able to measure the biological aging process more accurately than conventional chronological age [45,46]. From the 1960s, a rich body of evidence has demonstrated that the methylation status of more than 28 CpG sites might be associated with chronological age [26,33,47,48,49]. Investigating how endogenous and exogenous stressors affect the estimated epigenetic age across groups with a similar chronological age, for instance, could help identify anti-aging interventions [50]. Accordingly, different epigenetic “age estimators” have been proposed, as a combination of age-sensitive CpG sites and mathematical algorithms, to predict the so-called epigenetic clock of the DNA source, giving information on the aging status of single cells, tissues, or organs [23]. In 2018, Steve Horvath and Kenneth Raj described the epigenetic clock theory of aging and summarized the most important DNA methylation-based age estimators [23], which will therefore not be subject of this review. However, some of the most commonly used estimators in CRC research are summarized in Figure 1. Specifically, the Hannum’s clock was developed for the first time in 2013, as a 71 CpG-based epigenetic age estimator from blood DNA [51]. As Hannum’s algorithm was trained on whole blood from adults, it could return biased estimates in children and in non-blood tissue. To overcome this issue, Horvath and colleagues presented the first multi-tissue epigenetic age estimator, often referred to as Horvath’s clock, which is based on the methylation levels of 353 CpG sites [50]. Different from Hannum’s clock, this estimator was trained on 8000 microarray profiles of different tissue and cell types from children and adults. Although Hannum’s and Horvath’s clocks were correlated well with chronological age, they showed a weak association with clinical parameters. For this reason, Levine and colleagues developed the DNAm PhenoAge by regressing a set of ten clinical characteristics on DNA methylation data [52]. This estimator was based on 513 CpG sites and predicted mortality, risk for cardiovascular disease, and numerous indexes of multimorbidity well [52]. As for Hannum’s clock, however, the DNAm PhenoAge could provide biased estimates in children and non-blood tissues. Finally, Weidner and colleagues showed that an epigenetic age estimator, based only on three CpG sites, might predict chronological age with an absolute deviation of less than 5 years [53]. 

**Figure 2:**
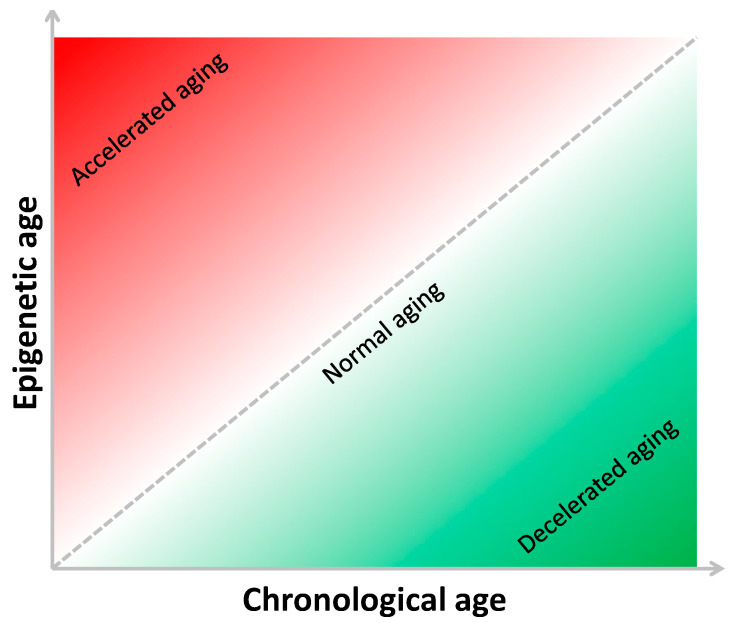
The relationship between chronological and epigenetic age.

The present review presents epidemiological studies investigating the association of epigenetic age, evaluated using different DNA methylation-based age estimators, with CRC risk and prognosis. In 2016, Zheng and colleagues evaluated the epigenetic age of 442 cancer-free participants from the U.S. Department of Veterans Affairs’ Normative Aging Study [54]. Specifically, they used Hannum’s 71-CpG method to estimate the epigenetic age in blood samples, and to predict biological age acceleration (Δage) as the difference between epigenetic and chronological age [54]. This concept and how it depends on the relationship between chronological and epigenetic ages are depicted in Figure 2. 

**Figure 3:**
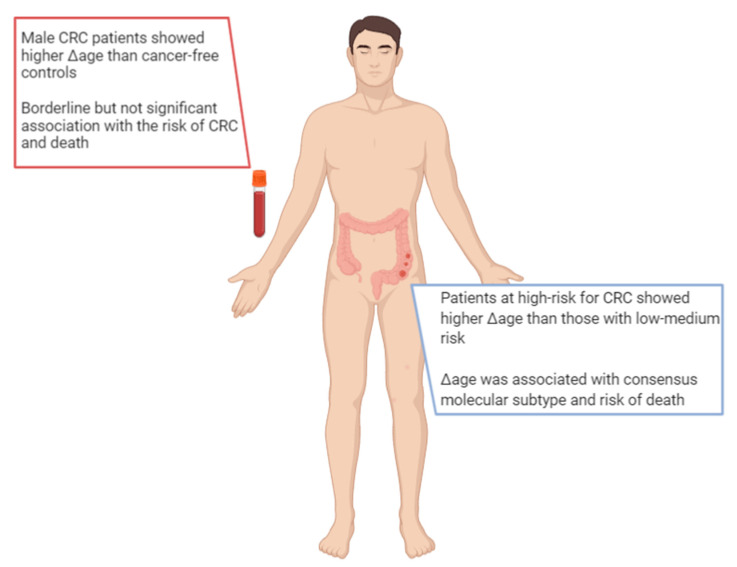
Main findings on epigenetic age in patients with colorectal cancer.

Interestingly, Δage was associated with cancer onset or death; as such, for a one-year increase in Δage, the risk increased by 6% and 17%, respectively. By contrast, people with decelerated biological aging (i.e., those with small or negative Δage) exhibited the lowest risk of cancer [54]. With respect to CRC, the evidence is still scarce, and researchers have only begun to examine this issue only in recent years (Figure 3). For instance, Durso and colleagues estimated and compared the epigenetic age between 166 Italian patients who developed CRC, and 425 participants who remained cancer-free over ten years of follow-up within the European Prospective Investigation into Cancer and Nutrition (EPIC) project [55]. In this case, they evaluated the epigenetic age of blood samples using three DNA methylation-based age estimators (i.e., Horvath 353-CpG, Hannum 71-CpG, and Weidner 3-CpG methods) and two specific age-sensitive genes (i.e., *elongation of very long chain fatty acids* (*ELOV2*) and *four and a half LIM domains protein 2* (*FHL2*)). Among women, the authors reported no differences in Δage between those who developed CRC and those who did not. Among men who developed CRC, instead, they noted a significantly higher Δage than their cancer-free counterparts: specifically, male CRC patients were 1.6 and 2.5 years older according to the Horvath’s and FHL2 clocks [55]. More recently, Dugué and colleagues aimed to assess the associations of Δage with risk for and survival from seven common cancers, including colorectal cancer [56]. They used Hannum’s and Horvath’s clocks to estimate epigenetic age and Δage in blood samples from 3216 cases and matched controls recruited by the Melbourne Collaborative Cohort Study. In general, the authors found that risk of cancer and death increased with increasing Δage (i.e., approximately 15–30% higher comparing the fourth versus first quartile of Δage). With respect to CRC, the analysis of 835 patients and their matched controls showed borderline, but not significant, associations with a risk of cancer and death [56]. 

The concept of epigenetic age and Δage could also apply to other sources of DNA, such as tissues and organs. In fact, other studies have estimated the epigenetic age of colonic mucosa to identify the factors that associated with biological aging, CRC risk, and specific molecular subtypes. For instance, Wang and colleagues analyzed the genome-wide DNA methylation profile of normal colon mucosa from 334 subjects, categorized at low, medium, or high risk for CRC, according to personal and clinical characteristics [57]. They used the Hannum’s, Horvath’s, DNAm PhenoAge, and EpiTOC methods to assess epigenetic age and Δage. Among all of the DNA methylation-based age estimators considered, Horvath’s clock exhibited the highest correlation coefficient with chronological age. According to DNAm PhenoAge, instead, patients at high-risk for CRC showed a higher Δage than those collocated in the other groups [57]. By contrast, Zheng and colleagues used Horvath’s clock to estimate the epigenetic age of colonic mucosa from 345 CRC patients and to investigate its association with other molecular features, clinical characteristics, and patients’ prognosis [58]. Interestingly, Δage was associated with consensus molecular subtype, a gene expression-based molecular classification established by the Colorectal Cancer Subtyping Consortium. Moreover, the authors demonstrated that Δage was positively associated with risk of death [58].

## 5. MiRNA Aging

It is worth mentioning that the aging process might also be characterized by different patterns of miRNA expression. The first evidence of this relationship came from Caenorhabditis elegans models, where the miRNA expression was related to lifespan and longevity [59,60,61]. Among more than 50 miRNAs differentially expressed during C. elegans aging, about half had conserved sequences in humans [60,62]. In particular, the majority of miRNAs were down-regulated [60,63], while only miR-34 was up-regulated during aging [60,63,64,65]. This was not consistent with the findings from studies in mice, where it an up-regulation of miRNA expression during aging was generally reported [66,67]. In fact, miRNAs might regulate tissue- and cell-specific aging phenotypes in mammals [68]. For instance, a comparison between young and old tissues in mice, primates, and humans showed that several miRNAs were differentially expressed during the aging process [68]. However, it is difficult to define a general and universal miRNA profile associated with aging, as changes in miRNA expression are generally tissue-specific. This is consistent with the current notion that aging signaling pathways play a different role depending on the type of tissue and organ.

As seen below, miR-34 has been extensively investigated in cancer research because it targets the genes involved in cellular senescence pathways [69,70]. Cellular senescence, in turn, can be induced and regulated by the tumor suppressor protein p53 in response to different stressors [71]. Specifically, p53 induces miR-34 expression, which targets and suppresses Sirtuin 1 (SIRT1) functions. The inhibition of SIRT1 mediated the deacetylation of tumor protein 53 (p53), and promoted its activities [69,70]. In the colon, the excessive proliferation of epithelial cells might result in their hyperplasia, and in some cases, prelude cancer. For this reason, miR-34 is often considered to be a tumor suppressor miRNA, and its expression was down-regulated in CRC specimens compared with normal mucosa [72]. Beyond miR-34, several studies have suggested the potential application of other miRNAs as biomarkers in CRC. The current state of the art of this field of research has been clearly summarized by previous reviews [39,73,74,75]. However, most of studies were conducted on small cohorts with heterogeneous patient populations and using non-established screening or diagnostic methods [73]. In fact, only a limited number of miRNAs (e.g., miR-21 [76] and miR-224 [77]) showed promising application in the clinical setting after meta-analytic studies. However, apart from them, miR-20a-5p, miR-103a-3p, miR-106a-5p, and miR-143-5p have been proposed as novel predictive markers for the recurrence of stage II CRC [78]. With respect to response to treatment, instead, the upregulation of miR-126 was associated with resistance to bevacizumab, whereas overexpression of miR-31, miR-100, miR-125b, and downregulation of miR-7, with resistance to cetuximab [78]. In 2018, an integrative analysis of colorectal cancer miRNA datasets identified 19 differentially expressed miRNAs that were directly associated with CRC through the interaction with the mismatch repair and signaling pathways [79]. 

In human blood samples, specific miRNA expression patterns are associated with age-related diseases, such as cancer [80,81] and cardiovascular disease [82]. More recently, the study by Huan and colleagues identified 127 miRNAs that were differentially expressed by age in more than 5000 participants of the Framingham Heart Study [83]. Specifically, the majority were under-expressed in older people. In turn, the miRNA levels were correlated with age-associated mRNA expression changes in the pathways related to RNA processing, translation, and immune function [83]. The authors also constructed an algorithm based on 80 miRNAs in order to estimate the so-called “miRNA age”, which might complement the predicted age from DNA methylation signatures. Interestingly, they found that accelerated aging (i.e., expressed as the difference between miRNA age and chronological age) was associated with all-cause mortality and cardiometabolic parameters [83]. However, to the best of our knowledge, no studies have investigated the association of miRNA age with CRC risk and prognosis yet.

## 6. Discussion

Since its discovery, epigenetics has been helping researchers to elucidate the molecular mechanisms underpinning the biological aging process and its association with the risk of age-related diseases, including cancer. In line with this, a growing body of evidence has already demonstrated that specific epigenetic modifications, such as aberrant DNA methylation and miRNA expression, are common in patients who develop CRC. As some of these signatures also characterize the biological aging process, several studies have investigated age-related changes in DNA methylation and miRNA expression that might be associated with the risk for and survival from CRC. Although aged and cancer tissues share some hypermethylated regions with a similar chromatin structure, it is not the same for hypomethylated regions that instead tend to differ [38]. Regarding the miRNA expression, miR-34 is currently the most extensively investigated in aging and cancer research, because of its involvement in cellular senescence pathways [69,70]. However, more robust findings are needed in order to explain how miR-34, as well as other miRNAs, might affect the aging process and the risk of age-related diseases. In fact, both DNA methylation and miRNA profiles are generally tissue-, organ-, and organism-specific, making it difficult to draw any convincing conclusion about the role of age-related epigenetic changes that might be also involved in cancer development [50]. In the framework of CRC research, there is a crucial need for novel diagnostic and prognostic biomarkers with a high specificity and sensitivity. The isolation of cancer-derived components (e.g., circulating tumor cells, tumor DNA, miRNAs, long non-coding RNAs, and proteins) from peripheral blood and other body fluids could lead to the so-called “liquid biopsy”. This represents a minimally invasive approach that could work alongside traditional techniques for screening and diagnosis, for predicting relapse and metastasis, and for monitoring chemotherapy resistance in CRC patients [74,84]. 

In this scenario, several estimators have been proposed to give an early indication of biological aging as the difference between epigenetic and chronological age [23,50]. The majority of them are based on the methylation level of specific sets of CpGs in blood samples. Accelerated biological aging, indicated by a high and positive value of Δage, was positively associated with a risk of cancer and death in general [54,83]. Yet, findings of these associations in the field of CRC are still scarce and, in some ways, controversial. Moreover, there is evidence of a sex-dependent effect of accelerated biological aging on CRC risk [55]. More recently, an algorithm for miRNA age has also been proposed, which consists of an estimator with a good ability to predict all-cause mortality and cardiometabolic risk [83]. However, the application of this novel concept is still in its infancy, and no studies on CRC have been conducted so far. Thus, there is still the need to investigate whether epigenetic age estimators, along with other biological aging markers (e.g., telomere length and frailty indexes), might be useful to identify future CRC patients before clinical symptoms arise [85]. 

An area that deserves deeper exploration is the assessment of epigenetic age directly in colonic mucosa; this might help to explain different physiological and pathological events that characterize CRC development in response to endogenous and exogenous stressors. In fact, there is high heterogeneity in the specific effect of genetic mutations that might influence the risk of CRC. Just to mention a few, the most common genetic mutations promote tumorigenesis by perturbing the function of key signaling pathways (e.g., WNT-β-catenin, epidermal growth factor, mitogen-activated protein kinase, phosphatidylinositol 3-kinase, and tumor growth factor β), or by affecting the genes that regulate DNA repair and proliferation [86]. 

Beyond genetic variations, environmental factors and behaviors, such as dietary habits [8,87] and physical activity [88], might modulate the risk of CRC. Thus, differences in CRC incidence among countries could be partially explained by these modifiable habits that vary according to sociodemographic and cultural conditions [89,90,91]. Interestingly, it has been estimated that nearly 70% of CRC cases could be avoided with a healthy lifestyle [92]. Although it is actually unknown if biological aging acceleration takes place as a consequence of events preceding the onset of CRC, some behaviors (e.g., cigarette smoking, unhealthy diet, and physical inactivity) have shown a moderate but significant effect on epigenetic age [2]. For this reason, the assessment of epigenetic age in people at risk for CRC could be helpful in monitoring the molecular response to preventive interventions [85]. Moreover, it would be interesting to investigate whether aberrant epigenetic aging could help identify CRC patients with a high risk of recurrence and a worse prognosis, as well as those who respond poorly to treatment [85]. To do all this, however, more efforts are recommended to standardize research protocols and to assess the cost-effectiveness of such estimators. Moreover, additional biomarkers should be considered in anticipation of future applications in clinical practice. 

In conclusion, our review points to the assessment of epigenetics as a promising topic for research into CRC. Despite interesting findings, there are still various limitations and pitfalls that need to be addressed before considering application in clinical practice. However, estimating the epigenetic age of patients at risk of CRC could be an important option in the pre-screening period, while its assessment in patients that have already developed a tumor could help with predicting their prognosis and/or monitoring their response to treatment. Thus, further large-size prospective studies using standardized protocols should be encouraged in order to make these perspectives a reality.

## Abbreviations

CRC	Colorectal cancer
WHO	World Health Organization
MUTYH	mutY DNA glycosylase
CpG	Cytosine-phosphate-guanine
miRNA	microRNA
CIMP	CpG island methylator phenotype
MLH1	MutL homolog 1
LINE-1	Long interspersed nuclear element 1
EPIC	European prospective investigation into cancer and nutrition
ELOV2	Elongation of very long chain fatty acids
FHL2	Four and a half LIM domains protein 2
SIRT1	Sirtuin 1
P53	Tumour protein 53
